# Basal Ganglia Involvement and Altered Mental Status: A Unique Neurological Manifestation of Coronavirus Disease 2019

**DOI:** 10.7759/cureus.7869

**Published:** 2020-04-28

**Authors:** Kaveh Haddadi, Roya Ghasemian, Misagh Shafizad

**Affiliations:** 1 Neurological Surgery, Orthopedic Research Center, Mazandaran University of Medical Sciences, Sari, IRN; 2 Infectious Diseases, Antimicrobial Resistance Research Center, Mazandaran University of Medical Sciences, Sari, IRN

**Keywords:** basal ganglia, altered mental state, covid-19, case report

## Abstract

Like other respiratory viruses, severe acute respiratory syndrome coronavirus 2 (SARS-CoV-2) may enter the central nervous system (CNS) via the hematogenous or neuronal path. However, neurological complications of coronavirus disease 2019 (COVID-19) have not been reported frequently. Encephalopathy has been described as a presenting symptom or complication of COVID-19 in some reports. We report a case of a 54-year-old patient who presented with unique clinical characteristics and imaging with brain basal ganglia involvement likely due to SARS-CoV-2 infection. In our experience, the incidence of spontaneous bilateral basal ganglia hemorrhage is rare. Further study will be needed to investigate this finding of the CNS and altered mental status in patients with this new type of coronavirus infection. Based on the case presented and other cases, understanding the pathways of virus neuroinvasion is necessary to help recognize possible pathologically related consequences of infection and to evaluate new diagnostic and management approaches that will help improve SARS-CoV-2 infection treatment and control.

## Introduction

Coronavirus disease 2019 (COVID-19) has become a pandemic. Whereas most prior studies focused on how the coronaviruses involve the respiratory system due to their typical manifestations in most patients, new research is providing a troubling indication that the new coronavirus can also involve the central nervous system (CNS) in different ways and cause short-term and long-term damage [1,2].

Neurological complications of COVID-19 have not been frequently reported. Encephalopathy has been described as a presenting symptom or complication of COVID-19 in some reports [1-6]. Here we report the case of a 54-year-old patient who presented with unique clinical and imaging with brain basal ganglia involvement most probably due to severe acute respiratory syndrome coronavirus 2 (SARS-CoV-2) infection.

## Case presentation

A 54-year-old woman with a past medical history of diabetes, hypertension, and a history of lumbar spinal laminectomy and fusion surgery three years ago was referred to our emergency department because of loss of consciousness. She had a low-grade fever (38°C) and cough for the past five days. Losartan and metformin were important recorded drugs in the patient’s drug history. Her primary vital signs were a blood pressure of 150/100 mmHg, pulse rate of 90 beats per minute, and oxygen saturation of 90%.

**Figure 1:**
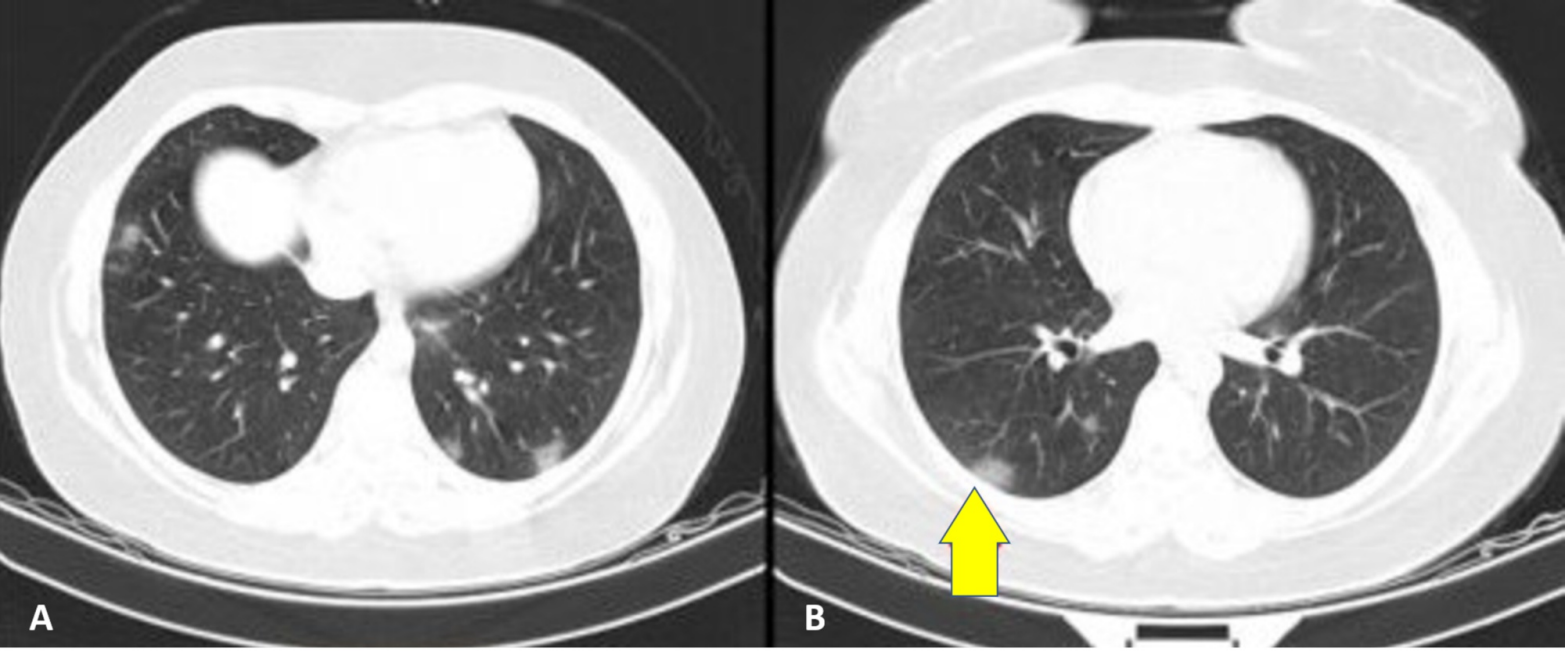
A lung computed tomography scan showing small diffuse patchy consolidation (A) and bilateral ground-glass opacities (B), characteristic of COVID-19 infection

The patient had a full laboratory workup and chest x-ray, the results of which were not revealing. The patient's Glasgow Coma Scale (GCS) score was about 10 at initial admission, and there was no trauma documented in the examination. Brain and lung computed tomography (CT) scans were taken, and the patient was admitted to the intensive care unit. All protective measures and precautions for a suspected COVID-19 infection were taken. The patient was placed in isolation. A lung CT scan showed small diffuse patchy consolidated bilateral ground-glass opacities (Figure 1).

**Figure 2:**
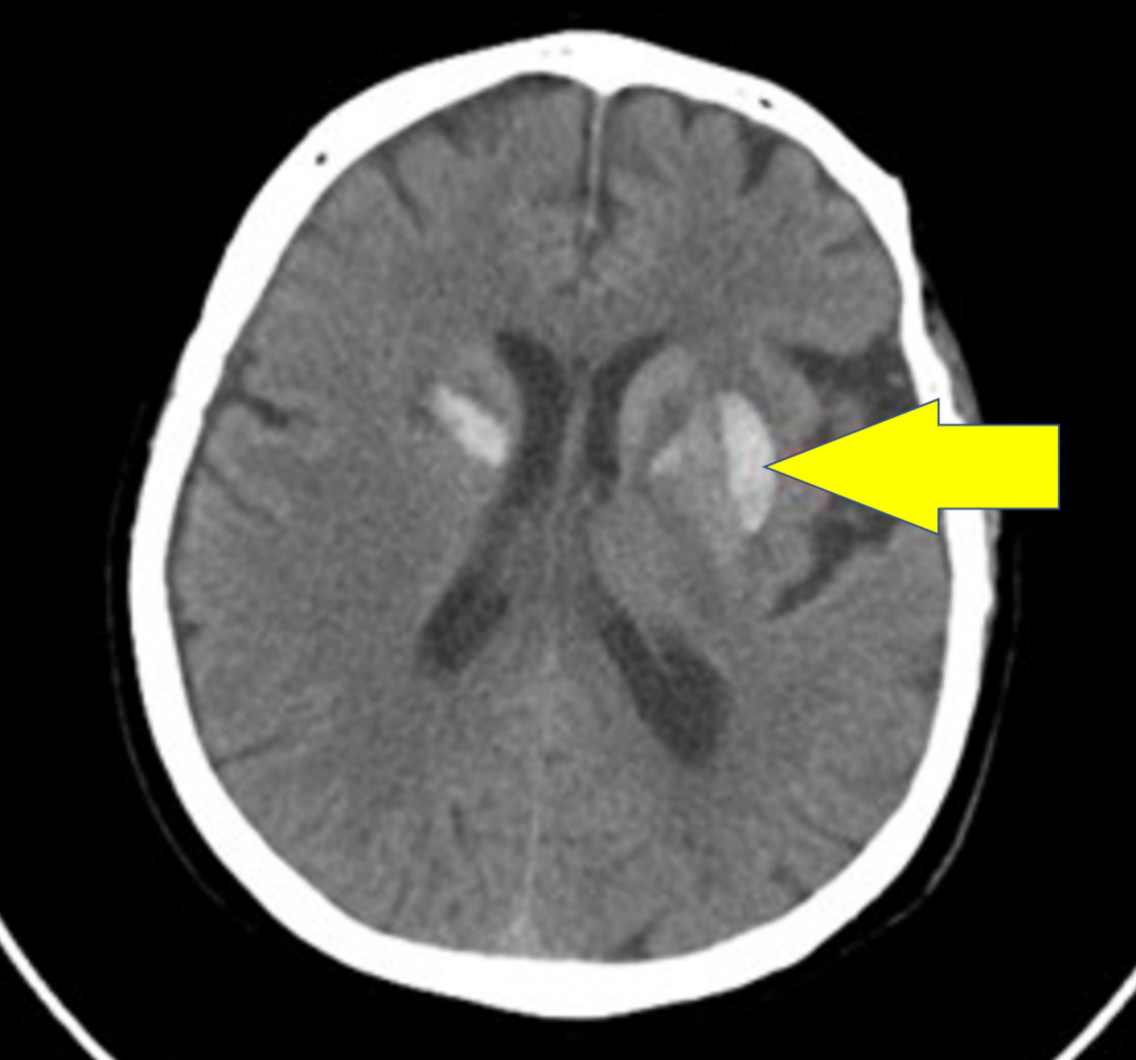
A computed tomography scan of the head that shows acute to subacute changes evident of bilateral basal ganglia hyperdensity matching with a subacute hemorrhagic event

A brain CT revealed bilateral basal ganglia hyperdensity matching a subacute hemorrhagic insult (Figure 2). 

**Figure 3:**
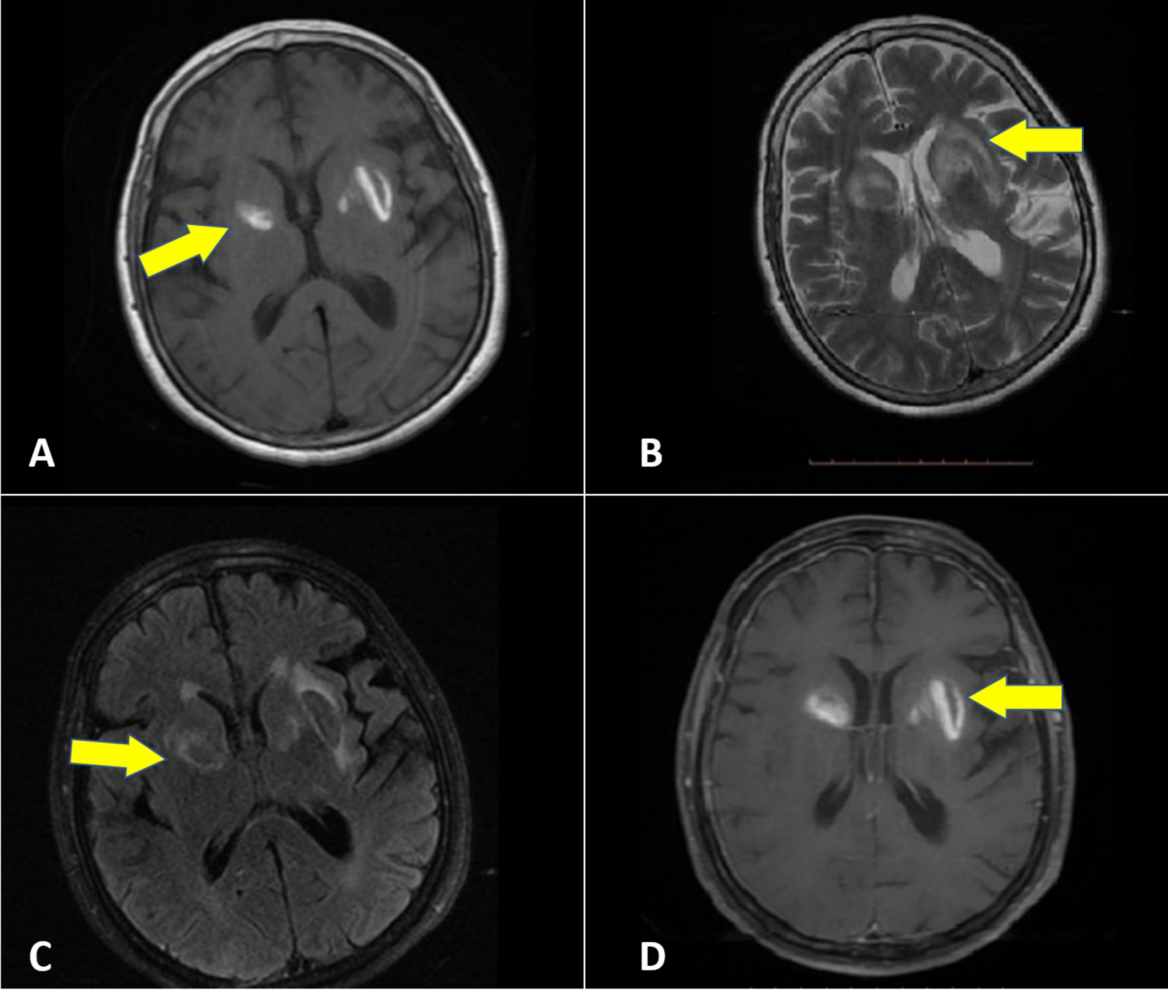
Magnetic resonance imaging T1 (A), T2 (B), fluid-attenuated inversion recovery (C), and gadolinium+ (D) showing the signal change in bilateral basal ganglia matching a bleeding event

Both throat sputum and nasopharyngeal cultures were tested for COVID-19. Blood cultures were negative, and urine analysis was negative. Brain magnetic resonance imaging (MRI) documented definite characteristics of basal ganglia involvement in subacute bleeding (Figure 3). 

Due to the severe alteration in mental status, a repeat examination was done. Upon examination, the patient was found to have encephalopathy-related disorders, was nonverbal, and unable to follow any orders; however, she was able to move all her extremities and was reacting to painful stimuli. No nuchal rigidity was noted. Her GCS score remained between 10 and 12.

Her blood glucose level was 250 mg/dL. All electrolytes were in the reference range. Test results for serum and urine ketone were negative. Thyroid function test results were normal, and the patient did not have anemia. Liver function tests were within reference ranges. White blood cell count was within the reference range. After two attempts to obtain cerebrospinal fluid (CSF), a lumbar puncture was abandoned as it was not possible due to previous lumbar surgeries and scarring.

The patient was started on antiepileptic medication prophylactically with phenytoin due to the possibility of convulsion. She was hydrated and treated with regular insulin based on her blood glucose levels and received routine antihypertensive drugs. Due to her progression in symptomatology, the patient was treated empirically with 400 mg hydroxychloroquine tablets.

Oral lopinavir/indinavir 200 mg twice daily, naproxen 250 mg twice daily, and levofloxacin 750 mg once daily were provided based on our infection department algorithm. Thankfully, after five days, the patient's vital signs and general condition stabilized, and she started to wake up.

Remarkably, the patient’s SARS-CoV-2 test was negative based on our laboratory policy and sample achievement. However, our final definitive diagnosis based on the lung CT scan and patient health scenario was COVID-19.

**Figure 4:**
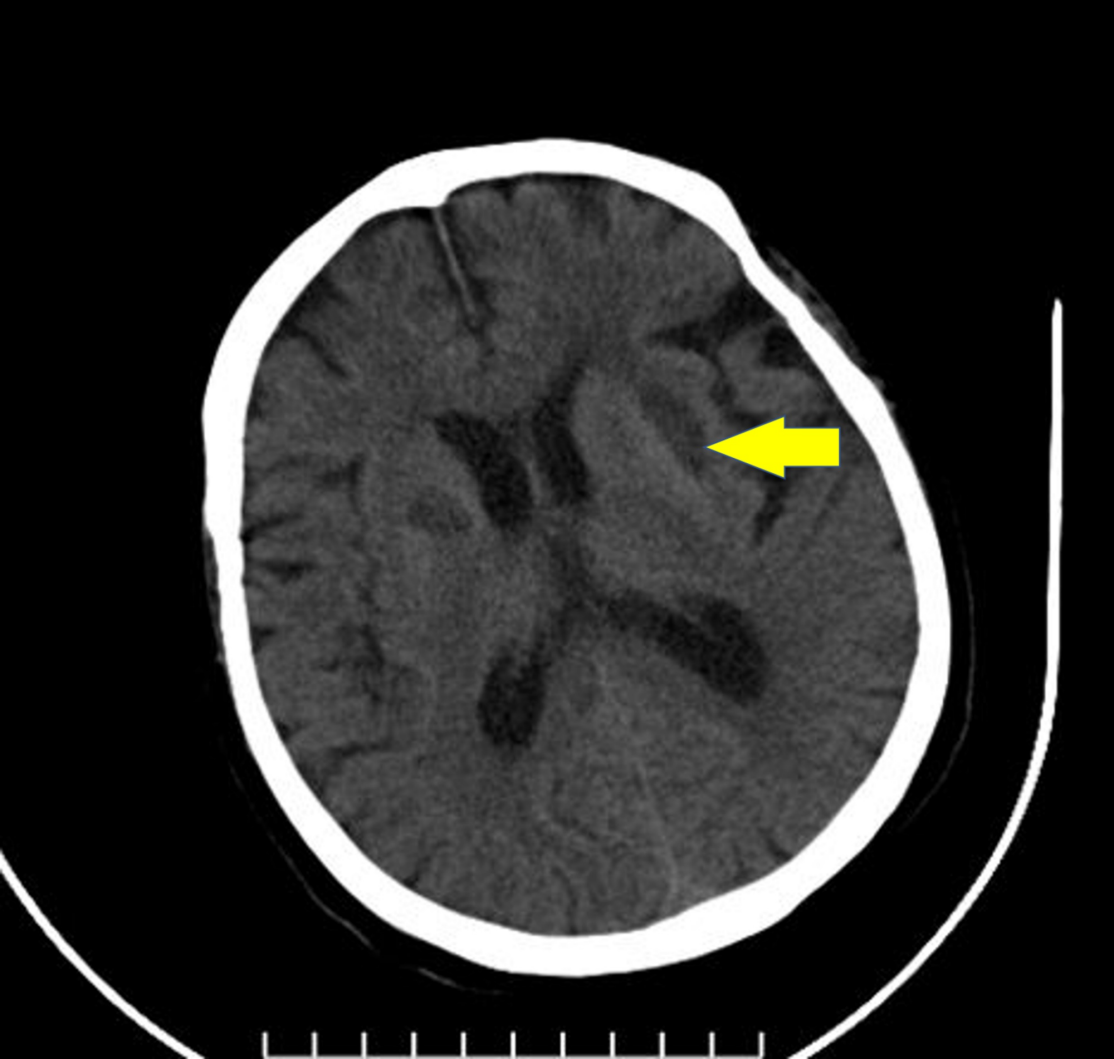
Follow-up brain computed tomography scan showing hypodensity after absorption of bleeding in the basal ganglia

On follow-up, the patient progressively improved in general. She was immediately mobilized on day 7, with good satisfaction. Her neurological, cognitive, and pulmonary functions had improved before she was discharged from the hospital. Brain CT scans performed in the first week after her release showed hemorrhagic absorption, and she was symptom-free at this period and returned to her normal duties (Figure 4).

## Discussion

The exact way in which SARS‐CoV or Middle East respiratory syndrome-related coronavirus (MERS‐CoV) enters the CNS has not been reported yet, but available data indicate that coronaviruses may first attack peripheral nerve terminals, and then gain entry to the CNS [7,8].

The entry of SARS‐CoV into human cells is facilitated by the angiotensin‐converting enzyme 2 (ACE2) cellular receptor, which is expressed in airway epithelia, lung, endothelia, kidney, and small intestine cells. However, the occurrence of ACE2 alone is not adequate to make host cells vulnerable to infection [7-10].

Similarly, SARS‐CoV‐2 possesses this same potential. Based on an epidemiological investigation on COVID‐19, the average time from the first symptom to dyspnea was five days, to hospital admittance was seven days, and to the intensive care unit was eight days. Then, the latency period might be enough for the virus to enter and abolish the medullary neurons [2].

Therefore, like other respiratory viruses, SARS-CoV-2 may enter the CNS via the hematogenous or neuronal path. The latter would be able to be supported by the information that some patients had hyposmia. The scientists identified SARS-CoV nucleic acids in the CSF of those patients and similarly in their brain tissue on autopsy [7-14].

On March 4, 2020, Dr. Liu Jingyuan disclosed that a 56-year-old patient with coronary pneumonia was confirmed by genetic sequencing to have SARS-CoV-2 in the CSF. His investigation found that the patient had presented specific conditions associated with CNS injury, but a CT scan of the patient's head did not reveal any abnormalities of the brain, and biochemical test results of the CSF were normal. However, diagnostic tests on the CSF of the patient uncovered the presence of the SARS-CoV-2, and the patient was treated for viral encephalitis [5].

On March 21, 2020, Asia Filatov et al. reported a 74-year-old man with respiratory disease, convulsion, altered mental status, confusion, and COVID-19 positive. Without CSF, however, doctors should be alert that patients with COVID-19 can present with encephalopathy in the acute phase [3].

We do not have a documented CSF analysis that confirms CNS infection. However, unlike the other reported cases with encephalopathy manifestations and normal brain CT scan, our patient had a typically positive brain imaging characterized by bilateral subacute (high T1 signal in MRI) hemorrhage in the basal ganglia that improved in further follow-up, matched with patient well recovery.

The basal ganglia and thalamus are paired deep gray matter structures that may be affected by a variety of diseases such as systemic or metabolic disease, degenerative disease, vascular conditions, and infections such as focal ﬂavivirus infections or toxoplasmosis. Radiology may detect bilateral abnormalities of the basal ganglia and thalamus in different acute (bleeding) and chronic clinical situations (calcifications), and, although MRI is the modality of choice for assessment, the accurate diagnosis can be completed only with all applicable clinical and laboratory information [15].

In our experience, the incidence of spontaneous bilateral basal ganglia hemorrhage is rare. Further investigation will be needed to generalize this finding of the CNS with altered mental status in patients with this new type of coronavirus infection.

During the epidemic period of COVID-19, when seeing patients with any neurological manifestations, health care providers should consider SARS-CoV-2 infection as a differential diagnosis to avoid delayed diagnosis or misdiagnosis and prevention of transmission.

## Conclusions

Based on the case presented and other cases, understanding the pathways of virus neuroinvasion is necessary to help recognize possible pathologically related consequences of infection and assist in the process of new diagnosis and management approaches that will help to improve the treatment and control of COVID-19.
